# Structural and Functional Studies of FKHR-PAX3, a Reciprocal Fusion Gene of the t(2;13) Chromosomal Translocation in Alveolar Rhabdomyosarcoma

**DOI:** 10.1371/journal.pone.0068065

**Published:** 2013-06-14

**Authors:** Qiande Hu, Yewen Yuan, Chiayeng Wang

**Affiliations:** 1 Center for Molecular Biology of Oral Diseases, University of Illinois at Chicago, Chicago, Illinois, United States of America; 2 Department of Oral Biology, University of Illinois at Chicago, Chicago, Illinois, United States of America; Virginia Commonwealth University, United States of America

## Abstract

Alveolar rhabdomyosarcoma (ARMS) is an aggressive pediatric cancer of skeletal muscle. More than 70% of ARMS tumors carry balanced t(2;13) chromosomal translocation that leads to the production of two novel fusion genes, PAX3-FKHR and FKHR-PAX3. While the PAX3-FKHR gene has been intensely studied, the reciprocal FKHR-PAX3 gene has rarely been described. We report here the cloning and functional characterization of the FKHR-PAX3 gene as the first step towards a better understanding of its potential impact on ARMS biology. From RH30 ARMS cells, we detected and isolated three versions of FKHR-PAX3 cDNAs whose C-terminal sequences corresponded to PAX3c, PAX3d, and PAX3e isoforms. Unlike the nuclear-specific localization of PAX3-FKHR, the reciprocal FKHR-PAX3 proteins stayed predominantly in the cytoplasm. FKHR-PAX3 potently inhibited myogenesis in both non-transformed myoblast cells and ARMS cells. We showed that FKHR-PAX3 was not a classic oncogene but could act as a facilitator in oncogenic pathways by stabilizing PAX3-FKHR expression, enhancing cell proliferation, clonogenicity, anchorage-independent growth, and matrix adhesion *in vitro*, and accelerating the onset of tumor formation in xenograft mouse model *in vivo*. In addition to these pro-oncogenic behaviors, FKHR-PAX3 also negatively affected cell migration and invasion *in vitro* and lung metastasis *in vivo*. Taken together, these functional characteristics suggested that FKHR-PAX3 might have a critical role in the early stage of ARMS development.

## Introduction

Chromosomal translocation is the most commonly observed genomic abnormality associated with hematological malignancies and sarcomas in humans. Most chromosomal translocations in cancer involve reciprocal exchange of DNA between two chromosomes, resulting in the formation of two novel fusion proteins [1]. Expression of both fusion gene products in tumor samples is infrequent. Typically, the fusion gene that inherits major functional domains from the parental proteins is consistently expressed and holds oncogenic property. By contrast, the reciprocal fusion gene is variably expressed (0-90%) and its function poorly understood. It is only recently that research, mainly in hematological cancers, suggests that the reciprocal fusion genes are more than passive byproducts of the translocations but rather active participants in the disease etiology. Several reciprocal fusion genes, mostly non-oncogenic themselves, can work in conjunction with the oncogenic fusion partners to promote oncogenesis and alter pathogenic specificity [2–6]. Collectively, these studies show that reciprocal fusion genes can contribute to human oncogenesis and that their variable expression pattern in patient-derived samples may not reflect their roles in the early stage of cancer development.

Rhabdomyosarcoma (RMS) is a heterogeneous group of malignant neoplasms of immature skeletal muscle. It is the most frequent type of soft tissue sarcoma found in children. The most common RMS is the embryonal type (ERMS) and the most aggressive is the alveolar type (ARMS). Over 75% of the ARMS are characterized by recurrent balanced chromosomal translocations [7–10]. The most prevalent is the t(2;13) (q35;q14) rearrangement that disrupts the transcription factor genes PAX3 on chromosome 2 and FKHR (FOXO1A) on chromosome 13. The translocation breaks within intron 7 of the PAX3 gene and intron 1 of the FKHR gene, leading to the formation of two fusion genes, PAX3-FKHR on derivative chromosome 13 and FKHR-PAX3 on derivative chromosome 2 [11,12]. The focus of the present study is on FKHR-PAX3, the reciprocal fusion gene of PAX3-FKHR that is linked to the development of ARMS.

Because high levels of PAX3-FKHR transcript and protein are detected in all t(2;13)-positive ARMS tumors and tumor-derived cell lines, and continuous expression of PAX3-FKHR is critical for maintaining ARMS phenotypes and cell survival [13], research efforts have focused exclusively on characterizing PAX3-FKHR gene products and their contribution to rhabdomyosarcomagenesis. The PAX3-FKHR fusion protein combines an intact PAX3 DNA binding domain (DBD) at its N-terminus to a bisected FKHR DBD and the intact FKHR activation domain (AD) at its C-terminus. This results in the formation of a powerful transcription factor with an enhanced transactivation activity [14,15] and broadened gene targets [16–21] compared to PAX3. Both *in vitro* transformation and *in vivo* tumorogenesis studies have strongly supported an active participation of PAX3-FKHR throughout the ARMS oncogenic process [22–25]. Despite these advances and breakthroughs, PAX3-FKHR driven rhabdomyosarcomagenesis remains difficult to model *in vivo*. Recent work has shown that PAX3-FKHR alone is incapable of driving ARMS development in mouse models unless accompanied by additional gene mutations. Some mutations that have been suggested to cooperate with PAX3-FKHR include inactivation of p53 and cdkn2d, and activation of Ras, N-Myc, and IRIZIO [26–29]. These mutations all lead to disruption in the p53 and Rb pathways. However, these mutations do not occur at high frequency in ARMS and most tumors derived from these pairings with PAX3-FKHR do not recapitulate the characteristic alveolar feature of the disease. Thus, it remains to be seen whether they are the natural cooperating partners of PAX3-FKHR or are acquired as secondary mutations during later stages of tumor evolution.

In contrast to PAX3-FKHR, the reciprocal FKHR-PAX3 fusion gene has received little attention in ARMS research primarily due to its inconsistent expression pattern. Although the FKHR-PAX3 genomic rearrangement is present in over 90% of tumor samples examined [30], FKHR-PAX3 transcripts are reported in only ~60-70% of the samples [30–32]. It is noteworthy that this frequency of expression is within the range (~60-95% [33]) reported for those reciprocal fusion genes that have recently been shown to carry leukemogenic functions. In view of these new developments on leukemia-associated reciprocal fusion genes and on the requirement for cooperating genetic events in PAX3-FKHR-driven ARMS, it seems premature to dismiss a biological contribution of FKHR-PAX3 in ARMS solely based on its expression pattern in well-established tumor samples. Thus, we have taken the first step towards characterizing FKHR-PAX3 fusion gene products and their potential role in oncogenic transformation and tumorogenesis. In this study, we isolated and cloned three FKHR-PAX3 isoforms from ARMS cells, and showed that FKHR-PAX3’s effects on various transforming phenotypes are suggestive of its involvement at the early stage of the disease development.

## Materials and Methods

### Materials

The reagents for PAX3-FKHR and CAT reporter constructs were as described [18,20,21]. The wild-type FKHR-GFP, triple-mutant FKHR-GFP and IGFBP1-Luc DNA constructs were provided by Dr. Terry Unterman (University of Illinois at Chicago). The pLKO-Tet-On DNA construct was provided by Dr. Marc Bissonnette (University of Chicago). Lentiviral DNAs and particles of plenti-EF1a-FKHR-PAX3-Rsv-GFP-Bsd and plenti-EF1a-(Null)-Rsv-GFP-Bsd were generated by AMS Biotechnology (Amsbio). Antibodies were purchased for MyoD and MyoG (BD Biosciences), myosin heavy chain and α-tubulin (Developmental Studies Hybridoma Bank), PAX3 (abcam), FAK (Santa Cruz), and FKHR, p-Y397, p-Y576/577, and p-Y925-FAK (Cell signaling). MG132 (carbobenzoxyl-leucyl-leucyl-leucinal) and 5’Aza-C (5-aza-2′deoxycytidine) were from Selleck-Chemicals.

### RNA, RT-PCR, qRT-PCR

Total RNA was prepared using Trizol reagent (Invitrogen). The cDNAs were prepared from DNase-treated RNA using First-strand cDNA synthesis kit (Fermentas). Quantitative PCR detection of gene expression was performed with QuantiFast SYBR Green with the rotor gene Q machine (Qiagen) using the following primers:

PAX3-FKHR:

5’GCACTGTACACCAAAGCACG3’(forward);
5’AACTGTGATCCAGGGCTGTC3’(reverse);

FKHR:

5’GCAGATCTACGAGTGGATGG3’(forward);
5’AACTGTGATCCAGGGCTGTC3’(reverse);

PAX3:

5’CAGCACCGTTCACAGACCTCA3’(forward);
5’CTAGTCTCTGACTGCAGCT3’(reverse);

FKHR-PAX3:

5’TACGCCGACCTCATCACCAAGGCCATCGA3’ (forward);
5’CTAGTCTCTGACTGCAGCT3’(reverse);

GAPDH:

5’CATGAGAAGTATGACAACAGCCT3’(forward);
5’AGTCCTTCCACGATACCAAAGT3’ (reverse)

Primers used in PCR/southern hybridization for detecting FKHR-PAX3 isoforms were:

5’AAGAGCAGCTCGTCCCGCCGCAAC3’(forward, F4);
5’TTGATACCGGCATGTGTGGCTTA3’(reverse, uPAX3c);
5’TTCAGAGCAGATTCTTCATATCTAG3’(reverse, uPAX3d);
5’TGGAATGTTCTAGCTCCTCG3’(reverse, uPAX3e);
5’AGAGCAGATTCTTCATATCTA3’(reverse, uPAX3g);
5’ATGTTTTGATATGTAACCATG3’(reverse, uPAX3h);

### DNA constructs

The full-length FKHR-PAX3 cDNAs from RH30 cells were isolated by RT-PCR using following primers:

5’ AGATCCCGTAAGTCGGGCGGCCTGGTA3’ (forward, uFK-2f);
5’ TTGATACCGGCATGTGTGGCTTA3’ (reverse, uPAX3c);
5’ TCAGAGCAGATTCTTCATATCTAG3’ (reverse, uPAX3d);
5’ ATGGAATGTTCTAGCTCCTCG3’ (reverse, uPAX3e).

The cDNAs containing only the open reading frame (ORF) sequence of FKHR-PAX3 isoforms were prepared using following primers:

5’ ATCTGGATCCGCCACCATGGCCGAGGCGCCTCAGGTGGTG3’ (forward);
5’ GATCTCGAGCTAAAAAGTCCAAGGCTTACT3’ (reverse, isoform c);
5’ GATCTCGAGTTACGCGATATCTGGCTTGAG3’ (reverse, isoform d);
5’ AGTACTCGAGTTATTGCTCCAGGTCTTCCTC3’ (reverse, isoform e).

All PCR generated DNA fragments were cloned into TOPO-TA vector for sequence verification. Sequence data of full-length FKHR-PAX3 isoforms c, d, and e have GenBank accession numbers of JX141474, JX141475, JX141476, respectively. The FP3-GFP construct was generated by in-frame joining of the FKHR-PAX3 ORF sequence upstream to the GFP sequence in the pEGFP-C1 vector using the BamHI/XhoI cloning sites. The PAX3-FKHR target-specific shRNA construct was generated by cloning the double stranded shRNA template oligonucleotides against the sequences surrounding the PAX3-FKHR fusion site (sense strand: 5’ CCGGGCCTCTCACCTCAGAATTCAATTCGTCATTTCAAGAGAATGACGAATTGAATTCTGAGGTGAGAGGCTTTTT3’; antisense strand: 5’ AATTAAAAAGCCTCTCACCTCAGAATTCAATTCGTCATTCTCTTGAAATGACGAATTGAATTCTGAGGTGAGAGGC3’) into the EcoRI/AgeI sites of the pLKO-Tet-On lentiviral vector DNA. Lentiviral particles were prepared from the 72 hour-conditioned media of 293T-17 cells that were transfected with a combination of pLKO-Tet-On lentiviral vector, gag, Rev, VSV-G DNA constructs.

### Cell culture

Murine C2C12 myoblasts and NIH3T3 fibroblasts, human RD ERMS, RH30 ARMS and 293T-17 embryonic kidney cell lines were purchased from ATCC. Human RH4 and RH28 ARMS cell lines were obtained from St. Jude Children’s Research Center [19]. All cell lines except C2C12 were maintained in Dulbecco's modified Eagle’s high glucose base medium supplemented with 10% fetal bovine serum (FBS). C2C12 myoblast cells were maintained in 15% FBS growth media and switched to 2% horse serum medium for myogenic differentiation [21]. Cells were replenished with fresh differentiation medium on a daily basis until end of experimentation. The FKHR-PAX3 or PAX3-FKHR stable expression clones were prepared from transfecting cells with pcDNA3-FKHR-PAX3 or pcDNA3-PAX3-FKHR DNA using lipofectamine method followed by a 14-day drug selection (400 μg/ml Geneticin). Early passage drug-resistant clones were expanded and frozen down within one week after final drug selection. To generate PAX3-FKHR and FKHR-PAX3 co-expressing cells, early passage PAX3-FKHR clones were infected with lentiviral particles carrying either control plenti-EF1a-(Null)-Rsv-GFP-Bsd or plenti-EF1a-FKHR-PAX3-Rsv-GFP-Bsd DNA followed by a 10-day drug selection (10 μg/ml blasticidin). The first confluent plate was designated as passage zero. To generate conditional PAX3-FKHR knockdown stable cells, ARMS cells were infected with lentiviral particles carrying pLKO-Tet-On DNA containing either scrambled or sh-PAX3-FKHR specific sequences followed by a 10-day drug selection (2 μg/ml puromycin). Conditional knockdown of PAX3-FKHR expression was achieved by treating cells with 10 μg/ml doxycycline (DOX). Routinely, cells were replenished with DOX containing medium every other day if experiment was to last more than two days.

### Immunodetection

Western blot analysis and immunofluorescence detection of MHC-positive C2C12 myotubes were carried out as previously described [21]. In brief, whole cell extracts were prepared in RIPA buffer (20 mM Tris-HCl, pH 8.0, 137 mM NaCl-, 1% NP-40, 10% glycerol, 10 μg/ml aprotinin, 10 μg/ml pepstatin A, 10 μg/ml leupeptin, 500 μM orthovanadate, 1 μM phenylmethylsulfonyl fluoride). Following SDS-PAGE, proteins were detected by chemiluminescent antibody detection kit (NEN Life Science). For visualizing MHC-positive myotubes by immunofluorescence, differentiated C2C12 cells were fixed with 1% paraformaldehyde and stained with anti-MHC antibody (MF20) followed by Alexa Fluor 488-conjugated secondary antibodies (Life technology). Afterwards, cells were counterstained with DAPI. Images were recorded using the Q-Capture Pro 5.0 image capture program (Leica DM/RB microscope).

### Promoter-reporter assays

Chloramphenicol acetyl transferase (CAT) and Firefly luciferase (Luc) reporter genes were used to measure transcription. Beta-galactosidase DNA (LacZ) driven by the CMV promoter was used for normalizing transfection efficiency. The CAT assay was as described [20], and Luc assay was carried out using the ONE-Glo™ Luciferase Assay (Promega). CAT and Luc activities were quantified by scintillation and luminometer, respectively.

### Clonogenic and Soft agar assays

For testing clonogenic (anchorage-dependent) function, cells were seeded in triplicate at a density of 5 x 10^3^ (RD and RH30) or 2.5 x 10^3^ (NIH3T3) cells/p100 mm dish and grown for 15 days with a single medium change at the mid-time point. Cells were fixed in 4% formalin and colonies were visualized with crystal violet blue staining. Soft agar assay was performed as previously described [34]. In brief, cells were seeded in triplicate at a density of 1 x 10^4^ cells/well in 0.3% Noble agar laid over a 2% Noble agar under-layer into six-well tissue culture plates. Fresh media were added every three days during colony growth. Quantitation was determined from counting colonies equal or greater than 200 μm in size from an average of five randomly selected fields per well. Images of colonies were captured using the MicroFire camera and PictureFrame application (Optronics).

### Cell adhesion, scratch wound, and invasion assays

All assays were performed as described [13]. In brief, matrix based adhesion assay was measured from seeding cells (2 x 10^4^ cells/well) that were suspended in 1% BSA containing serum-free into a 48 well-microtiter plate pre-coated with BSA and designated extracellular matrix (ECM) elements (Cell Biolabs). Cells were allowed to attach for one hour before rinsing with PBS to remove unattached cells. Attached cells were fixed, stained with crystal violet solution, and released from plates for quantitative absorbance analysis at OD590 nm. Trypsinization assay was measured by treating triplicate 6-well plates of exponentially growing cells with 250 μl of trypsin at variable concentration for 30 sec followed by shaking for 1 min. The detached cells were collected after adding 500 μl serum-free DMEM to the wells. The remaining cells were treated with another 250 μl of trypsin until all the cells detached and harvested in the same manner. Cells were stained with trypan blue and counted. Number of dead cells was negligible. The adhesion index was determined as % of total cells that detached after the initial trypsin treatment. Wound closure activity was measured from confluent cells (2 x 10^5^ cells/well) in a 12-well culture plate. Cells were seeded one day before the surface was uniformly scratched with a pipette tip across the center of the well. The initial wound area and the movements of the cells into the scratched area were captured on CCD Spot camera attached to a Leica Digital Microscope (DM2500). Quantitation of movement was calculated as the percentage of the mean distance of leading edges of migrated cells over the mean distance of the initial wound edges. Invasion activity was determined at 24 hours (RH30) and 48 hours (RD) post-seeding. Cells were incubated in serum free medium for 6 hours prior to being trypsinized and seeded in triplicate (2 x 10^4^ cells/insert seeded, Cell Biolabs) into control or Matrigel inserts. The lower chamber was filled with growth medium. After 24 or 48-hour incubation, non-migrating cells from the upper membrane were removed using wet Q-tips. Migrating cells attached to the underside of membrane were fixed, stained with crystal violet, and counted. Quantitation was calculated as percentage of the mean number of cell migrating through Matrigel membranes over the mean number of cells migrating through control membranes.

### *In vivo* tumor assay

Xenograft tumor induction was performed on 4-6 week-old male athymic nude mice (Harlan). RD vs. RD–FKHR-PAX3 or RH30 vs. RH30-FKHR-PAX3 cells (3 X 10^6^ cells/50 μl PBS) were injected intramuscularly into the hind leg muscle (n=10 per group). The tumor diameter was recorded in two dimensions upon first sign of nodule formation. Tumor volume was calculated using V=0.52x a x b^2^ formula where a and b are the long and short diameter of the tumor, respectively. At the end-point of experiment, the mice were sacrificed and tumors and vital organs were excised and stored for further analysis. A board certified pathologist (Dr. Joel Schwartz, UIC) evaluated all the primary and secondary tumor pathology in this study.

### Statistical analysis

The values represent mean ± s.d. of a minimum of three independent experiments. The s.d. is the root mean square deviation of the *n-1* determinations. The Student’s t-test was used to obtain the statistical significance with *p*< 0.05. *p*Value: * *p* < 0.05, ** *p* < 0.001.

### Ethics statement

This study was carried out in strict accordance with the recommendations in the Guide for the Care and Use of Laboratory Animals of the National Institutes of Health. The Office of Animal Care and Institutional Biosafety committee at the University of Illinois at Chicago approved the protocol (number: A11-041). The animals were sacrificed by cervical dislocation after sedation with CO_2_ from a bottled gas source, and all efforts were made to minimize suffering.

## Results and Discussion

For the past two decades, significant progress has been made linking the molecular consequences of t(2:13) translocation to ARMS initiation, progression, and maintenance. While PAX3-FKHR is proven to be necessary and contributes to many pathogenic features of ARMS, it is not the sole one to drive the disease formation. A major challenge in understanding ARMS etiology is the identification of the accompanying genetic events for PAX3-FKHR-induced rhabdomyosarcomagenesis. A promising candidate could be FKHR-PAX3, the reciprocal fusion gene formed at the same time as PAX3-FKHR. In this study, we characterized the expression and regulation of FKHR-PAX3 gene products in ARMS cells, and evaluated their role in the *in vitro* cellular transformation and *in vivo* tumorogenesis processes. Our results demonstrate that FKHR-PAX3 contributes to cell transformation process associated with early phases of tumorogenesis, thereby supporting FKHR-PAX3 as a potentially critical biological factor in ARMS pathogenesis.

### Cloning and expression of rhabdomyosarcoma FKHR-PAX3 reciprocal fusion gene

**Figure 1:**
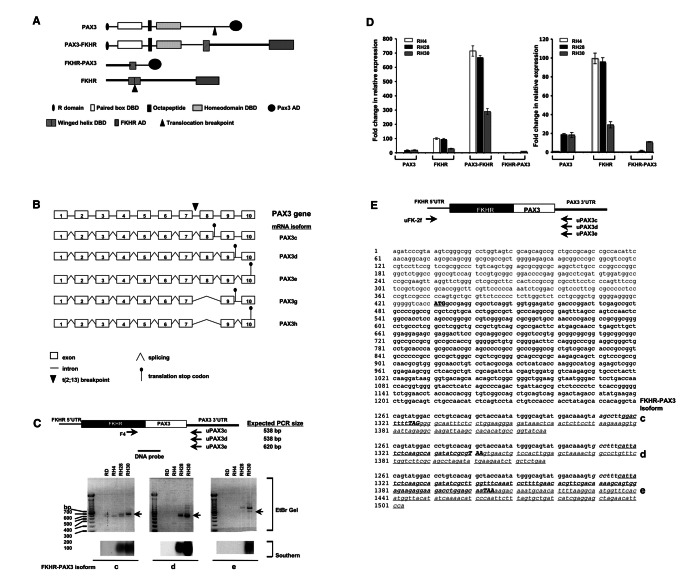
Cloning of FKHR-PAX3 cDNA. (**A**) Schematic of PAX3, FKHR, PAX3-FKHR and the predicted FKHR-PAX3 protein structures indicating the known functional domains. R: repressor; DBD: DNA binding domain; AD: activation domain. (**B**) Diagrammatic illustration of the exon-intron organization of human PAX3 gene, and the five alternatively spliced mRNAs that could result from processing of the FKHR-PAX3 primary transcript. PAX3c, PAX3d, and PAX3e use stop codons in intron 8, intron 9, and exon 10. PAX3g and PAX3h are truncated isoforms of PAX3d and PAX3e, respectively, that splice out exon 8. (**C**) Expression of FKHR-PAX3 transcript isoforms c, d, and e in ERMS (RD) and ARMS (RH4, RH28, RH30) cell lines as detected by RT-PCR and confirmed by Southern hybridization. Top panel: schematic indicates the positions of the FKHR-specific primer (F4) and the isoform-specific PAX3 PCR primer pairs, and the DNA probe spanning the FKHR-PAX3 fusion site used in the Southern analysis are indicated (not to scale). (**D**) Quantitative RT-PCR analysis of PAX3, FKHR, PAX3-FKHR, and FKHR-PAX3 expression in ARMS cell lines. The relative expression data are presented at two different scales on the Y-axis, high (left panel) and low (right panel) to compensate for the high levels of PAX3-FKHR expression. The relative expression level of PAX3/GAPDH in RH4 cells was assigned an arbitrary value of 1, and used as the reference to calculate fold change. (**E**) Nucleotide sequences of the cloned FKHR-PAX3 isoforms c, d, and e cDNAs. Top panel: schematic of the FKHR and the isoform-specific PAX3 primer pairs used to clone the full-length protein coding cDNA from RH30 cells. Primer location is approximate for illustrative purposes only. Sequence data is annotated by text in: Plain: 5’ FKHR-UTR; plain/italic: 3’ PAX3-UTR; Bold: protein coding sequences; bold/capital/underline: translation start codon; bold/capital/italic/underline: translational stop codons; underline: isoform-specific sequences.

The FKHR-PAX3 fusion joins the 5’-portion of the FKHR gene to the 3’-portion of the PAX3 gene. The fusion protein is predicted to combine the bisected FKHR DBD at its N-terminus with the intact PAX3 AD at its C-terminus (Figure 1A). Previous surveys detected low level of a FKHR-PAX3-specific RT-PCR product in approximately 60-70% of the t(2;13) ARMS tumor samples [30–32]. However, these studies did not evaluate transcript structure or protein expression. Transcript structure is of special interest because there are seven alternatively spliced PAX3 isoforms (a, b, c, d, e, g, h) with divergent C-termini [35–37]. The translocation breakpoint in PAX3 gene lies within intron 7, suggesting that the primary FKHR-PAX3 transcript could undergo alternative splicing to produce five potential isoforms (c, d, e, g and h; Figure 1B).

To evaluate this, we performed RT-PCR using a FKHR forward primer (F4) paired with the isoform-specific 3’ UTR PAX3 primer in four RMS lines, three t(2;13) positive ARMS (RH4, RH28, and RH30) and one ERMS (RD, Figure 1C, top and middle panels). Because of low expression and non-specific PCR products, we verified that the detection of FKHR-PAX3 mRNAs by southern hybridization using a DNA probe that spanned the fusion site (Figure 1C, bottom panel). FKHR-PAX3 isoforms c and d were detected in RH28 and RH30 cells whereas isoform e was detected only in RH30 cells. Despite repeated attempts, we were unable to detect FKHR-PAX3 isoforms g and h in any ARMS line (data not shown). This is perhaps not surprising because PAX3 g and h isoforms are primarily produced in melanocytes [37]. FKHR-PAX3 c and d are the predominant isoforms in ARMS, a finding that is consistent with the major PAX3 variants present in normal muscle and RMS cells [38]. Results from qRT-PCR analysis that compared the total amount of FKHR-PAX3 transcripts to those of PAX3, FKHR, and PAX3-FKHR in ARMS cells showed that the FKHR-PAX3 mRNA levels were within the same order of magnitude as PAX3 or FKHR (Figure 1D, right panel). In effect, all three genes, PAX3, FKHR, and FKHR-PAX3 were weakly expressed relative to the supraphysiologic PAX3-FKHR levels characteristic of ARMS cells (Figure 1D, left panel).

As expected, ERMS RD cells did not express FKHR-PAX3. The absence of FKHR-PAX3 transcript in RH4 cells is in agreement with the findings that this gene transcript is not detected in all t(2;13) ARMS tumors [30–32]. However, we do not believe that the variability in FKHR-PAX3 expression equates to its lack of function in ARMS pathogenesis. This point is particularly significant if FKHR-PAX3, like its reciprocal counterparts of leukemic cancer, is only needed early in the oncogenic process, a condition that no longer exists in the cancer cells of an established tumor.

We obtained cDNA clones containing full-length open reading frames of the three FKHR-PAX3 isoforms from RH30 cells by PCR amplification using the combination of a 5’-UTR of FKHR forward primer corresponding to sequence proximal to the FKHR transcription start site and an isoform specific 3’-UTR PAX3 primer (Figure 1E, schematic on top). DNA fragments of expected FKHR-PAX3 sizes were extracted from gel, cloned, and a minimum of three independent clones of each isoform type was sequenced. The DNA sequence of all three FKHR-PAX3 cDNA isoforms contained coding and non-coding sequences as predicted from the parent FKHR and PAX3 genes (Figure 1E).

**Figure 2:**
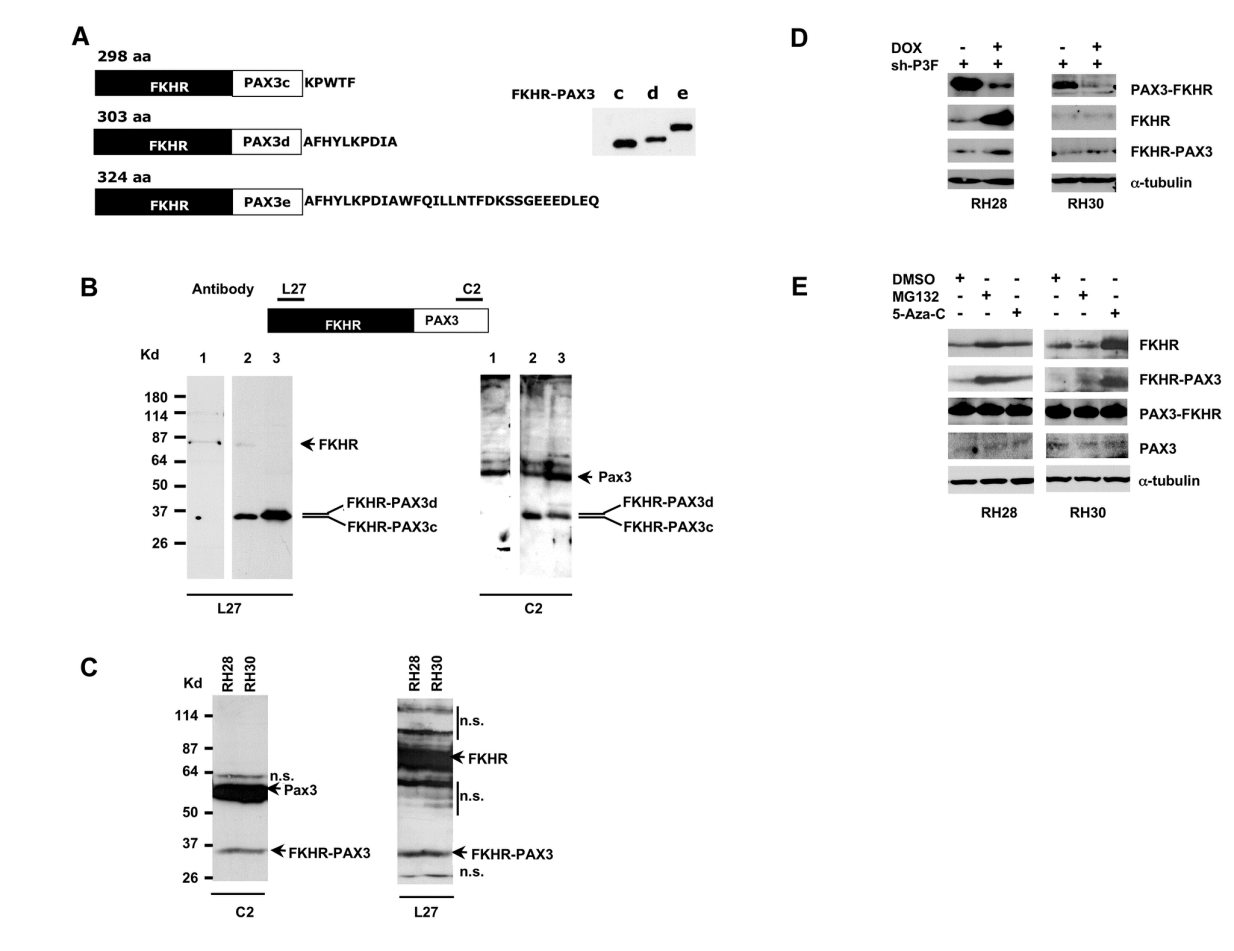
Immunodetection of in vitro and *in vivo* expressed FKHR-PAX3 protein. (**A**) Left panel: schematic of deduced sizes and amino acid sequence variations for FKHR-PAX3 isoforms c, d, and e. Right panel: Autoradiographic image of S^35^-methionine labeled *in vitro* translated FKHR-PAX3 protein isoforms. (**B**) Verification of PAX3-specific C2 and FKHR-specific L27 antibodies in detecting *in vivo* expressed FKHR-PAX3 proteins. Top panel: diagrammatic illustration of the epitope locations within the FKHR-PAX3 protein recognized by L27 and C2 antibodies. Bottom panel: western blot detection of FKHR-PAX3 in whole cell extracts (30 μg) prepared from RD cells that were transiently transfected with control expression vector (lane1), FKHR-PAX3 isoform c (lane 2), and FKHR-PAX3 isoform d (lane 3) using C2 (left panel) and L27 (right panel) antibodies. (**C**) Western blot detection of the endogenously expressed FKHR-PAX3 in RH28 and RH30 cells by L27 and C2 antibodies. Protein extract from FKHR-PAX3 negative RH4 cells was included as negative control. n.s.: non-specific bands resulting from high amount of protein extracts used and long film exposure. (**D**) Effect of PAX3-FKHR knockdown on the endogenous level of FKHR-PAX3 in RH28 and RH30 cells. Whole cell extracts were prepared from cells that stably expressed the inducible PAX3-FKHR shRNA treated with DMSO or DOX for 48 hours, and analyzed for FKHR and FKHR-PAX3 expression. (**E**) Effect of MG132 (10 μM for 12 hours) and 5’-Aza-C (1 μM for 48 hours) on endogenous FKHR-PAX3 expression levels in RH28 and RH30 cells. (**C**-**E**) A total of 400 μg of protein extracts were used for the analyses. (**D**-**E**) Alpha-tubulin was used to normalize sample loading.

The DNA sequence of all three FKHR-PAX3 isoforms c, d, and e had open-reading frames of 298, 303, and 324 amino acids (Figure 2A, left panel), and produced *in vitro* translated proteins of observed 35, 36, and 38 kd molecular weights on SDS-PAGE analysis (Figure 2A, right panel). Because FKHR-PAX3 specific antibody was not available, we used two commercial antibodies, L27 against a N-terminal FKHR epitope and C2 against a C-terminal PAX3 epitope, to detect *in vivo* FKHR-PAX3 proteins in cell extracts. We validated that these antibodies could detect ectopically expressed FKHR-PAX3 isoforms c and d in ERMS RD cells (Figure 2B). Subsequent analysis of RH28 and RH30 cell extracts confirmed the presence, albeit at low levels, of endogenous FKHR-PAX3 protein; the protein was undetectable in RH4 cells that did not express the FKHR-PAX3 mRNAs (Figure 2C). Because *in vivo* expressed FHKR-PAX3 may or may not be post-translationally modified in cells, the endogenous FKHR-PAX3 band detected in ARMS cells could contain more than one protein isoform. It should be noted that the amount of extracts needed to detect FKHR-PAX3, FKHR, and PAX3 proteins was over 20-times higher than the amount needed to detect PAX3-FKHR under the same conditions. The pattern and levels of PAX3, FKHR, FKHR-PAX3, and PAX3-FKHR protein are consistent with their relative RNA levels in these cells (Figures 1D and 2C).

**Figure 3:**
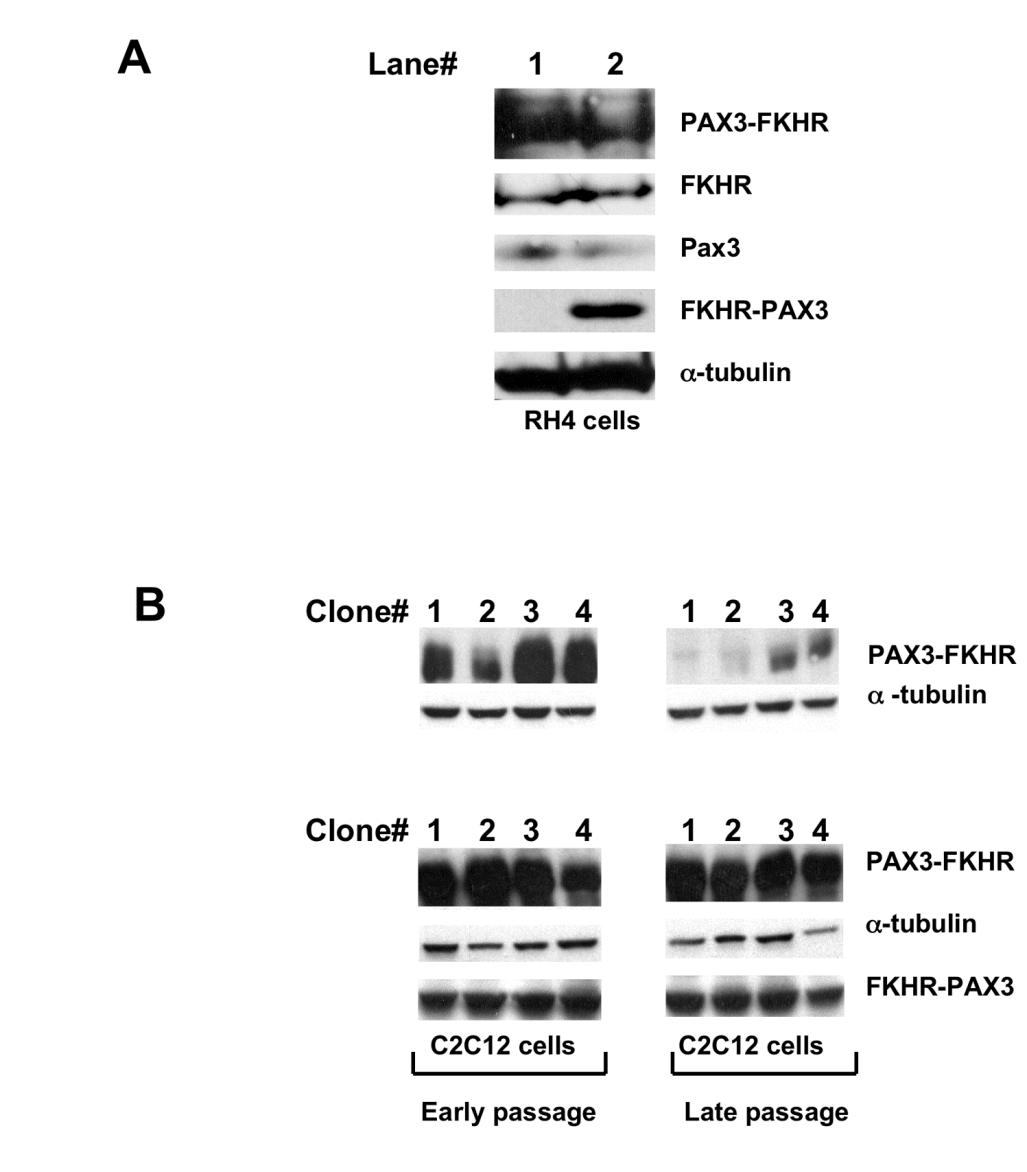
FKHR-PAX3 preserved high level of PAX3-FKHR expression in myogenic cells. (A) RH4 cells that do not express endogenous FKHR-PAX3 were transfected with empty vector (lane 1) or FKHR-PAX3 expression vector (lane 2). After continuous culture for 35 passages, cells were assayed for PAX3, FKHR, PAX3-FKHR, and FKHR-PAX3 expression by western blot. (B) Western blot analysis on the ability of FKHR-PAX3 to sustain high PAX3-FKHR expression. Stable clones of C2C12 cells expressing high levels of PAX3-FKHR were subjected to a second round of transfection to select for FKHR-PAX3 expression as described in Materials and Methods. The first confluent plate was designated as passage zero and used to generate early (< 10) and late (>35) passage cells. Cells were cultured at comparable density and passaged every three days. Immunoblot images were from four representative control and FKHR-PAX3 expressing lines. L27 antibody was used to detect FKHR-PAX3, and α-tubulin was used to normalize sample loading. Representative data from FKHR-PAX3 isoform c is shown.

Because ARMS cells preferentially expressed PAX3-FKHR at high levels, we wondered whether the large amount of PAX3-FKHR protein might interfere with FKHR-PAX3 expression, thus contributing to a diminished level of FKHR-PAX3 observed in these cells. To test this, we transfected a tetracycline-inducible shRNA to knockdown PAX3-FKHR expression in RH28 and RH30 cells. As shown in Figure 2D, doxycycline (DOX) treatment drastically reduced PAX3-FKHR but not FKHR-PAX3 protein levels in both cell lines. Interestingly, PAX3-FKHR knockdown led to a significant increase of FKHR expression in RH28 but not RH30 cells. The precise reason for this differential response is unknown. It could be related to the genetic heterogeneity in the different tumor cell lines, a notion supported by data in Figure 2E. In an effort to identify other pathways regulating FKHR-PAX3, we performed a small-scale chemical screen focusing on pharmacological agents that are known to regulate FKHR expression. Although most showed no effect, we did find two agents, 26S proteasome inhibitor MG132 and hypomethylating agent 5’ Aza-C, up-regulated both FKHR and FKHR-PAX3 expression in these ARMS cells. The effects of these agents were cell line specific, with RH28 cells responding to MG132 and RH30 cells responding to 5’ Aza-C. The result of 5’ Aza-C indicates that FKHR gene may be hypermethylated in RH30 cells, thus accounting for the differential response of PAX3-FKHR knockdown in the two ARMS cell lines. However, the most intriguing observation is that FKHR-PAX3 responds similarly as FKHR to signals that are part of normal cell function but differently from FKHR to pathogenic signal such as PAX3-FKHR. While future analysis on additional cell lines will be needed to confirm these regulatory patterns, the current data seem to suggest a need for cells, possibly around the time of gene fusion, to maintain FKHR-PAX3 expression. This idea seems to be supported by the following studies when we examined the effects of FKHR-PAX3 on PAX3-FKHR expression (Figure 3).

### FKHR-PAX3 promoted high levels PAX3-FKHR expression in myogenic cells

Despite the disparity in the expressed levels between the two fusion proteins in established cancer cells, we cannot exclude the possibility that FKHR-PAX3 could have an effect on PAX3-FKHR expression, for example, in early developing cancer cells. To investigate this possibility, we tested if high level of FKHR-PAX3 would affect PAX3-FKHR expression in the context of both transformed and non-transformed muscle cells. As shown in Figure 3A, we did not detect a change in the PAX3-FKHR protein content in RH4 cells when ectopically expressing FKHR-PAX3 to a level equal to that of PAX3-FKHR. Of note, knockdown of endogenous FKHR-PAX3 expression in RH28 and RH30 cells did not change PAX3-FKHR expression nor affect the growth behavior and survival of these cells (data not shown). Instead, we found that FKHR-PAX3 significantly increased the number of non-transformed C2C12 myoblast clones with sustained high PAX3-FKHR expression (Figure 3B). This is an especially intriguing result because recapitulating an ARMS-like high PAX3-FKHR expression in non-transformed cells in long-term cultures has been difficult. High expressers either succumb to growth arrest and cell death or revert into low expressers after several passages [39,40]. Furthermore, the surviving low expressers were incapable of forming tumors in xenograft models. A recent study reveals that a co-oncogenic factor such as N-myc can prevent loss of PAX3-FKHR high expressers in human muscle progenitor cells and more significantly, only the high expressers can develop ARMS-like tumors in a xenograft mouse model [29]. Results from Figure 3B suggests that FKHR-PAX3 could substitute for the effect of N-myc in allowing high PAX3-FKHR expression in tumor progenitor cells that subsequently undergo clonal expansion and acquire additional mutations that promote cancer progression. Perhaps, the ability of FKHR-PAX3 to escape a negative feed back regulatory loop from PAX3-FKHR as observed in Figure 2D is critical for these early tumorogenic events.

### FKHR-PAX3 localized in cytoplasm and lacked transcription regulatory activity

**Figure 4:**
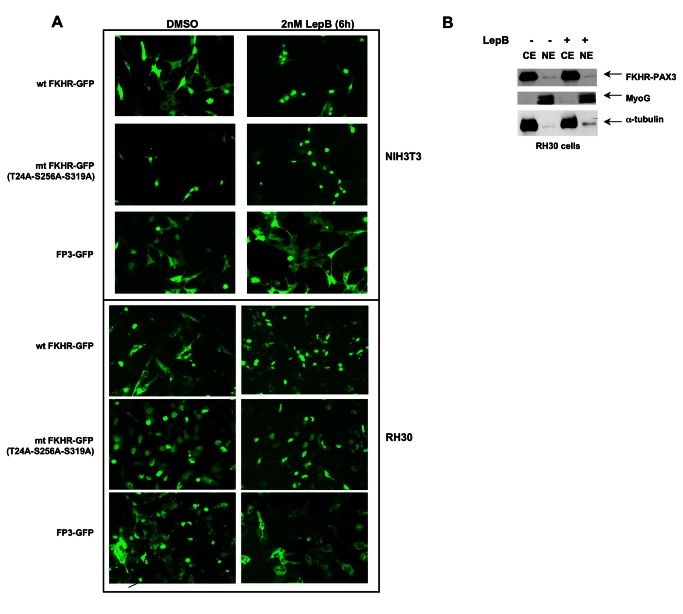
FKHR-PAX3 localized predominantly in the cytoplasm of cells. (**A**) NIH3T3 and RH30 cells were transfected with vectors expressing wild-type FKHR-GFP, triple-mutant (T24A/S256A/S319A) FKHR-GFP, or FKHR-PAX3-GFP (FP3-GFP) by lipofection. Cells were maintained in low serum medium (0.5% FBS) overnight prior to the addition of DMSO or LepB (2 μM) for six hours. At the end of treatment, fluorescent microscopy (magnification: 200X) was used to visualize the GFP-tagged proteins as indicated. (**B**) Western analysis confirmed the cytoplasmic localization of the FKHR-PAX3 protein in cells. RH30 cells were transfected with FKHR-PAX3 and treated with or without LepB as described in (**4A**). MyoG and α-tubulin served as nuclear and cytoplasmic specific controls, respectively, to evaluate the fractionation protocol. L27 antibody was used to detect FKHR-PAX3. Representative data from FKHR-PAX3 isoform c is shown.

PAX3 and PAX3-FKHR transcription factors are exclusively nuclear whereas FKHR transcription activity is tightly regulated through shuttling between nucleus and cytoplasm in response to stimuli. All confirmed nuclear localization signals (NLS) of PAX3 and FKHR, and a nuclear export signal (NES) of FKHR are retained in PAX3-FKHR, implying that FKHR-PAX3 might be unable to shuttle across the nuclear membrane. To test this, we created a chimeric FP3-GFP to visualize FKHR-PAX3 localization in live cells, and compared it to the behavior of two GFP-tagged FKHR controls: a wild-type FKHR-GFP whose nuclear export can be blocked by Leptomycin B (LepB), and a nuclear export defective triple-mutant FKHR-GFP with alanine replacing amino acids T24, S253 and S319 [41]. As shown in Figure 4A, in the absence of LepB, wild-type FKHR-GFP and FP3-GFP resided primarily in cytoplasm whereas the triple mutant stayed exclusively in the nucleus. In the presence of LepB, wild-type FKHR-GFP accumulated in the nucleus as expected whereas FP3-GFP remained mostly in the cytoplasm. The cytoplasmic-specific localization of FP3-GFP was confirmed by western blot using fractionated cytoplasmic and nuclear preparations (Figure 4B). Alpha-tubulin and MyoG were used as control markers for the cytoplasmic and nuclear fractions, respectively. The low level of FP3-GFP detected in the nuclear fraction was most likely due to contamination from the cytoplasmic fraction as a similarly low level of α-tubulin was also detected.

**Figure 5:**
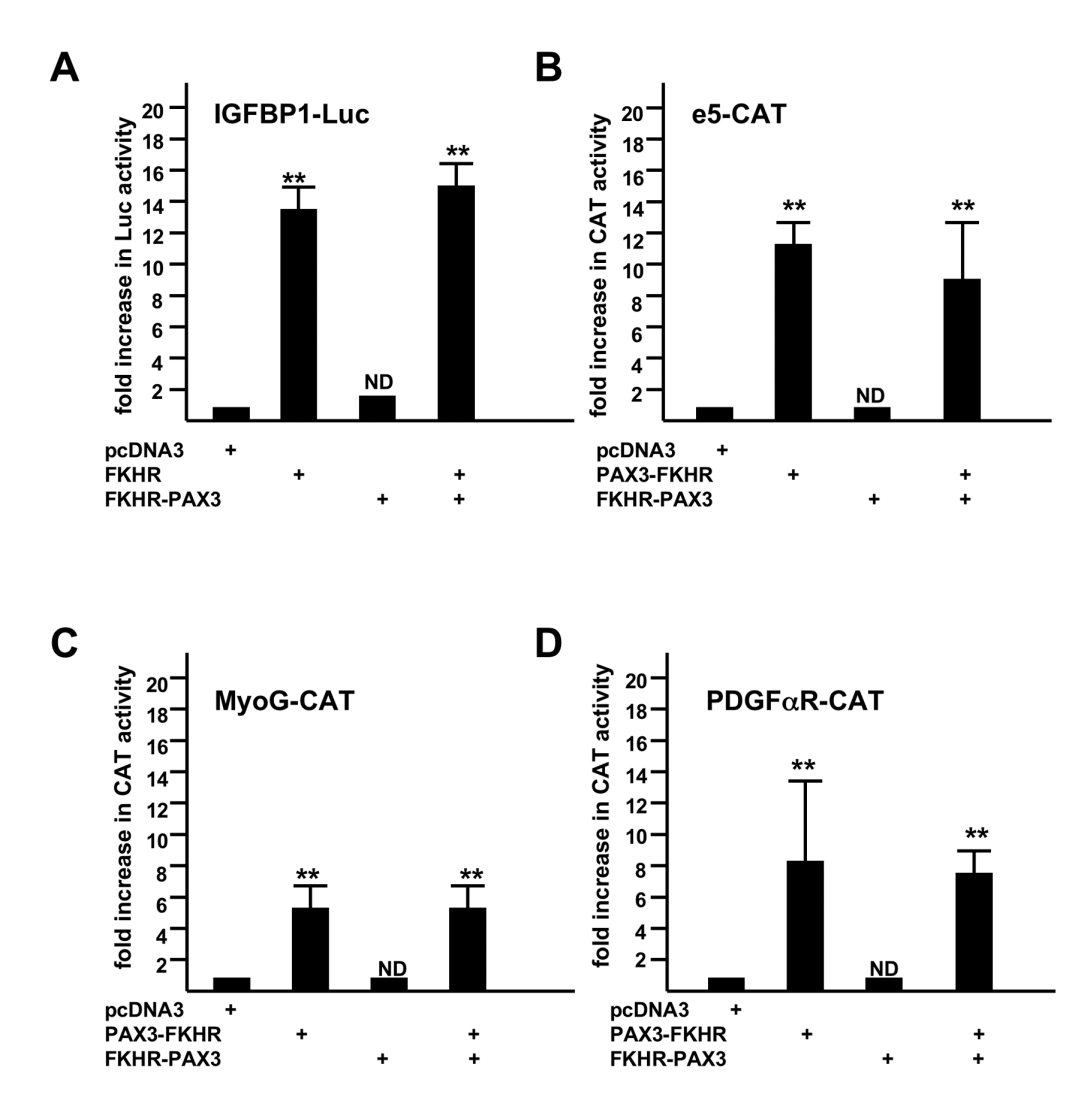
FKHR-PAX3 did not transactivate promoters that were responsive to FKHR (IGFBP1, A) and PAX3-FKHR (e5, B; MyoG, C; PDGFαR, D). C2C12 cells were transiently transfected with a total of 2 μg of DNA including 0.1 μg of LacZ, 0.5 μg of promoter-reporter (Luc or CAT as indicated), and 0.2 μg of the pcDNA3 vector or vector expressing the transcription factor by lipofection. After 48 hours, cells were harvested for LacZ, Luc, and CAT assays. Fold increase was calculated as the ratio of reporter activity from cells expressing the indicated transcription factor to the activity in cells transfected with the empty expression vector. Results were normalized to LacZ activity. The reporter activity in the presence of empty expression vector was assigned a value of 1. ND: statistically no difference. Representative data from FKHR-PAX3 isoform c is shown.

**Figure 6:**
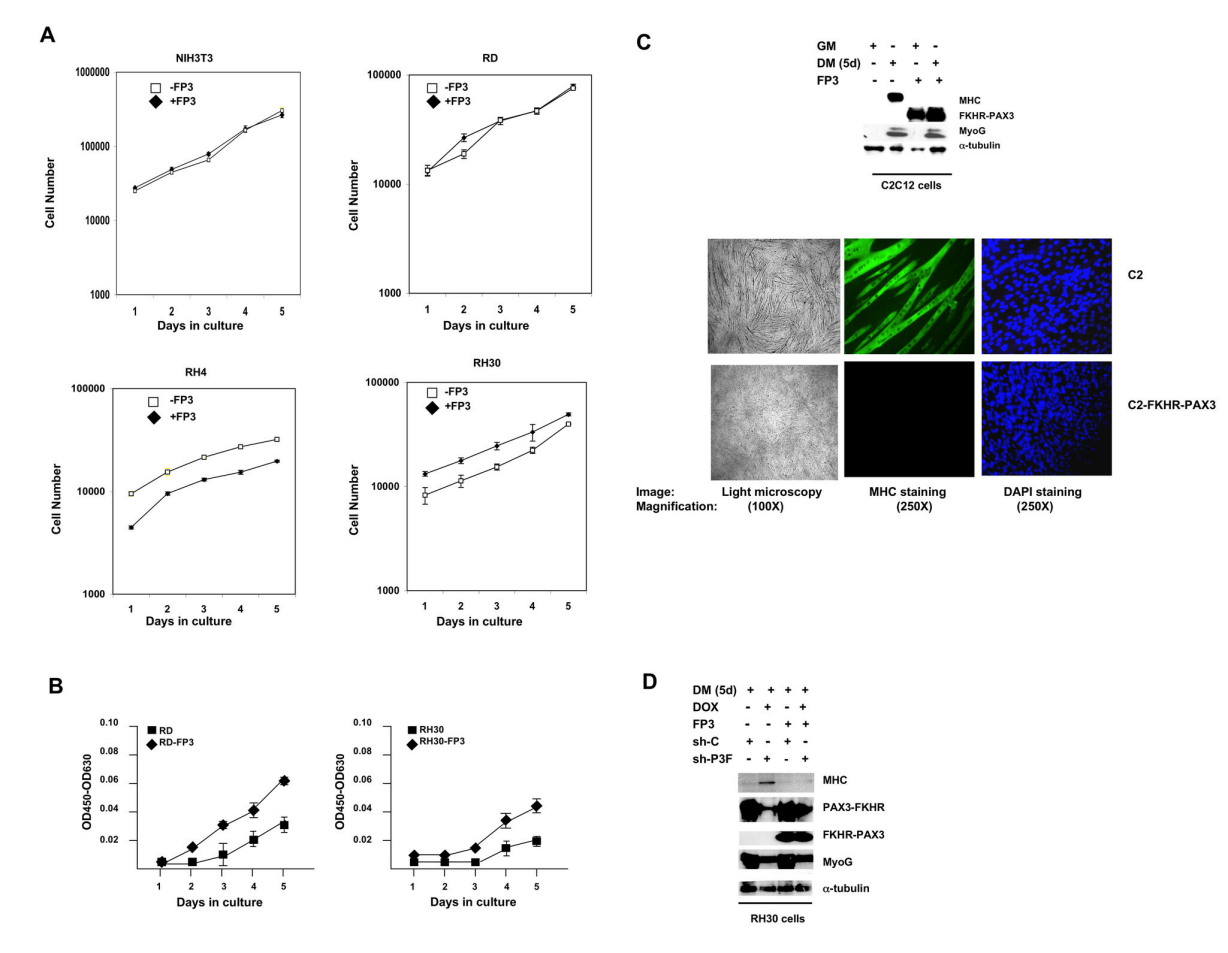
FKHR-PAX3 promoted low-density cell proliferation and blocked terminal myogenic differentiation. (**A**) Comparison of cell proliferation in NIH3T3, RD, RH4, and RH30 cultures with or without FKHR-PAX3 (FP3) expression. Cells were seeded in triplicate at 1 x 10^4^ cells/well into a 24 well-plate. Proliferation was measured daily by counting the number of live cells (trypan blue-negative) over five days beginning a day after the initial seeding. Cell death was minimal in all experiments. (**B**) The effect of FKHR-PAX3 expression on low-density RD and RH30 growth. Cells were seeded in triplicate at 2 x 10^3^ cells/well into a 24 well-plate. Cell growth was quantified daily using the WST-1 cell proliferation kit. (**C**) Top panel: Immunodetection of MyoG and MHC expression in proliferating (GM) and differentiated (five days, DM) C2C12 cells with or without FKHR-PAX3. Bottom panel: light (left panel, 100X magnification) and fluorescent (middle and right panels, (250X magnification) microscopic images of day-5 differentiated cells stained with MF20 antibody against MHC (middle) or with DAPI (right). (**D**) The effect of PAX3-FKHR knockdown on MyoG and MHC expression in control and FKHR-PAX3 expressing RH30 cells. Cell extracts were analyzed by western blot as in Figure 2D. (**C**-**D**) Alpha-tubulin was used to normalize sample loading. Representative data from FKHR-PAX3 isoform c is shown.

**Figure 7:**
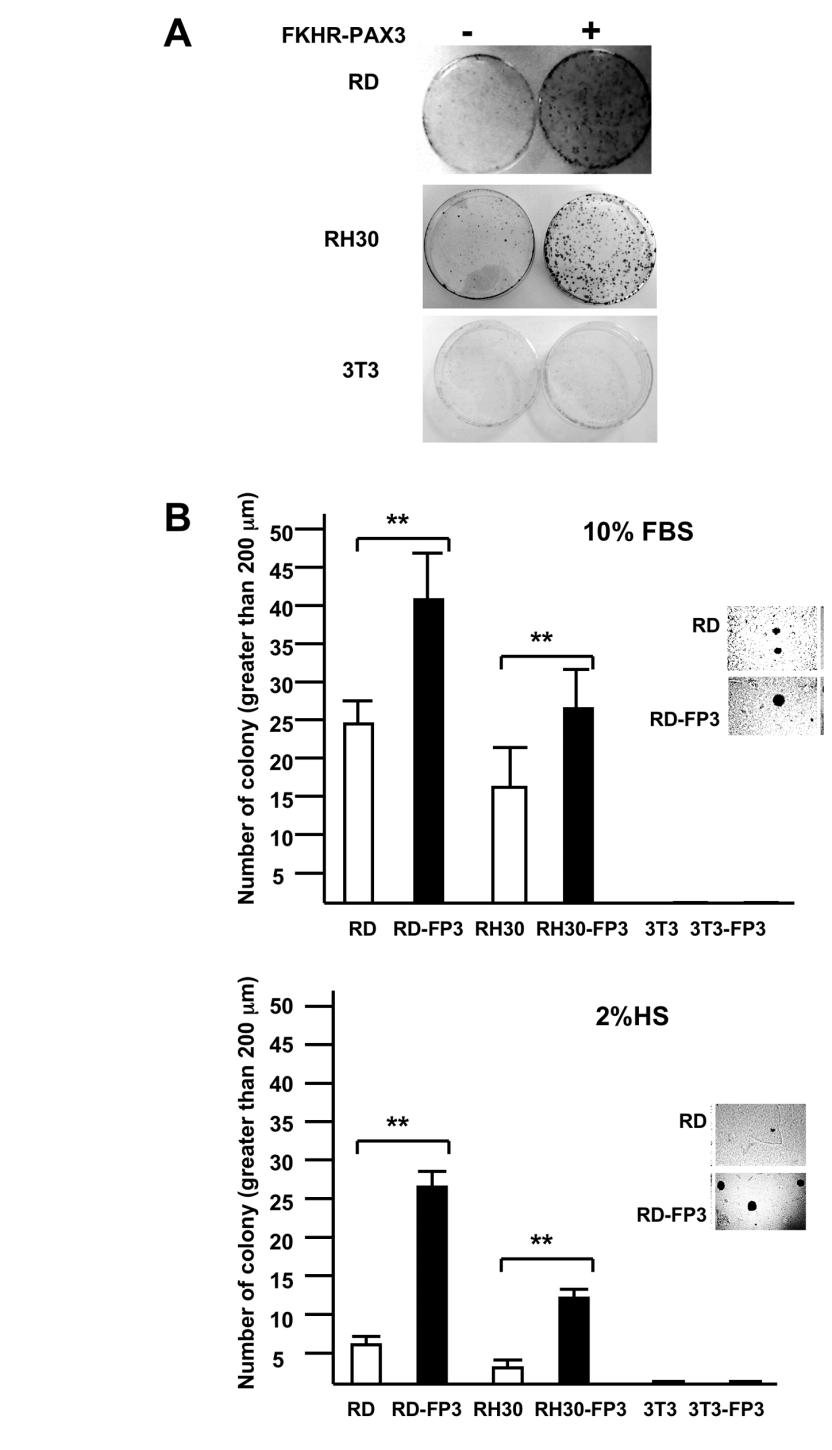
FKHR-PAX3 enhanced anchorage-dependent and anchorage-independent colony growth. (**A**) Photographic images of crystal violet blue stained cell colonies in the clonogenicity assay at the end of a 15-day growth (10% FBS). (**B**) Quantitative analysis of anchorage-independent soft agar colony formation under growth (10% FBS, top panel) and differentiation (2% HS, bottom panel) conditions. Inset: representative micrographic images of RD cells showing that FKHR-PAX3 (isoform c) enhanced both number and size of the colonies. (**A**-**B**) Assays were conducted as described in Materials and Methods. Representative quantitative data from FKHR-PAX3 isoform d is shown.

**Figure 8:**
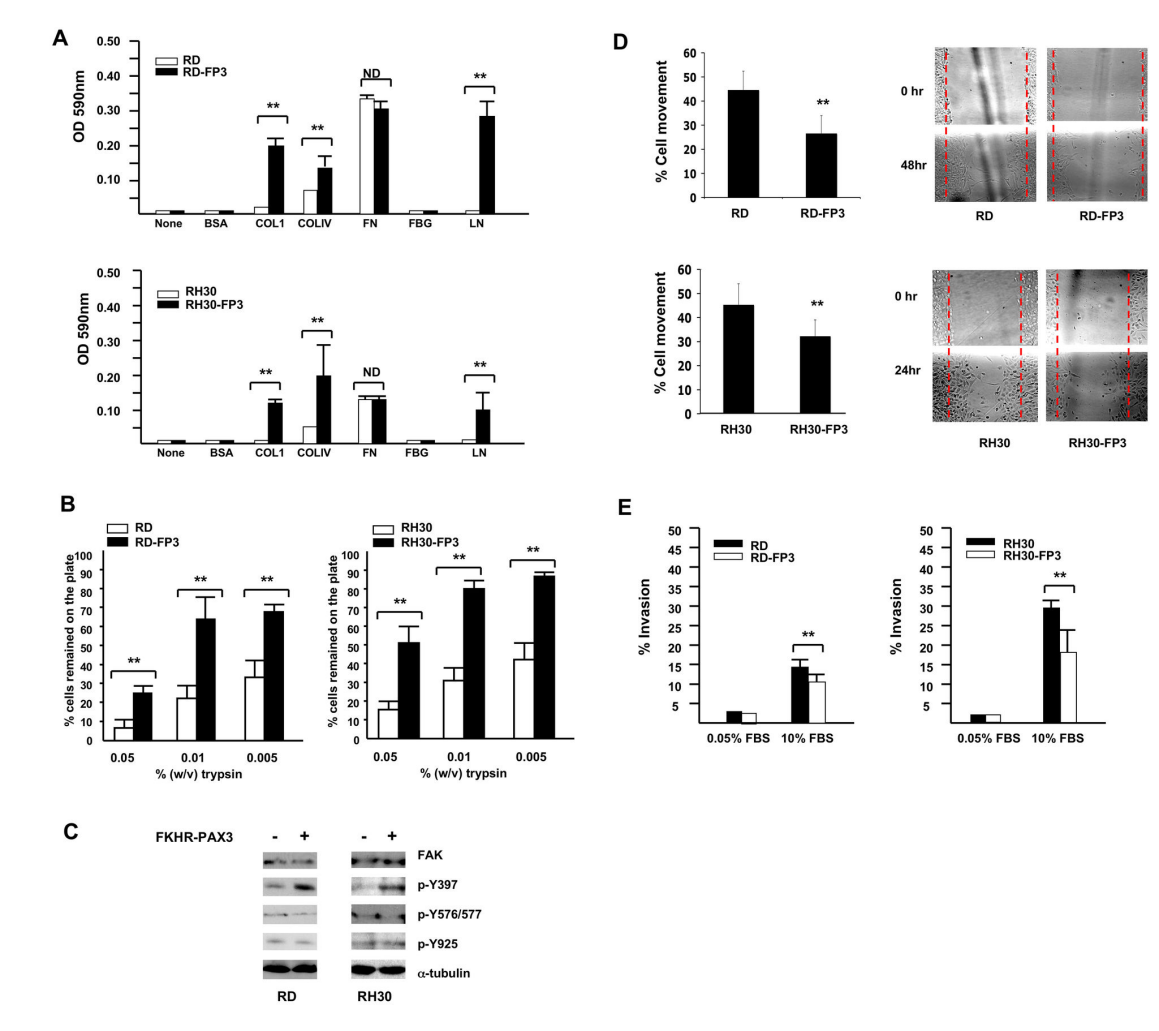
FKHR-PAX3 selectively increased cell adhesion over cell movement and invasion. (**A**) FKHR-PAX3 increased attachment of RD (top panel) and RH30 (bottom panel) cells to the indicated extracellular matrix components. (**B**) FKHR-PAX3 expression in RD (left panel) and RH30 (right panel) increased the strength of cell adhesion to culture dishes as indicated by a reduced sensitivity to trypsin. (**C**) Western blot analysis of the effect of ectopic FKHR-PAX3 expression on FAK phosphorylation in RD and RH30 cells. A total of 30 μg of whole cell extracts were analyzed. (**D**) FKHR-PAX3 reduced the migratory function in RD (top panel) and RH30 (bottom panel) cells as measured by scratch wound assay. Left panel: quantification of migratory index; right panel: representative micrographs of the scratched wound assays. (**E**) FKHR-PAX3 decreased the invasive potential of RD (left panel) and RH30 (right panel) as determined by Matrigel assay. ND: statistically no difference. (**A**-**B**, **D**-**E**) Assays were conducted as described in Materials and Methods. Representative data from FKHR-PAX3 isoform c is shown.

**Figure 9:**
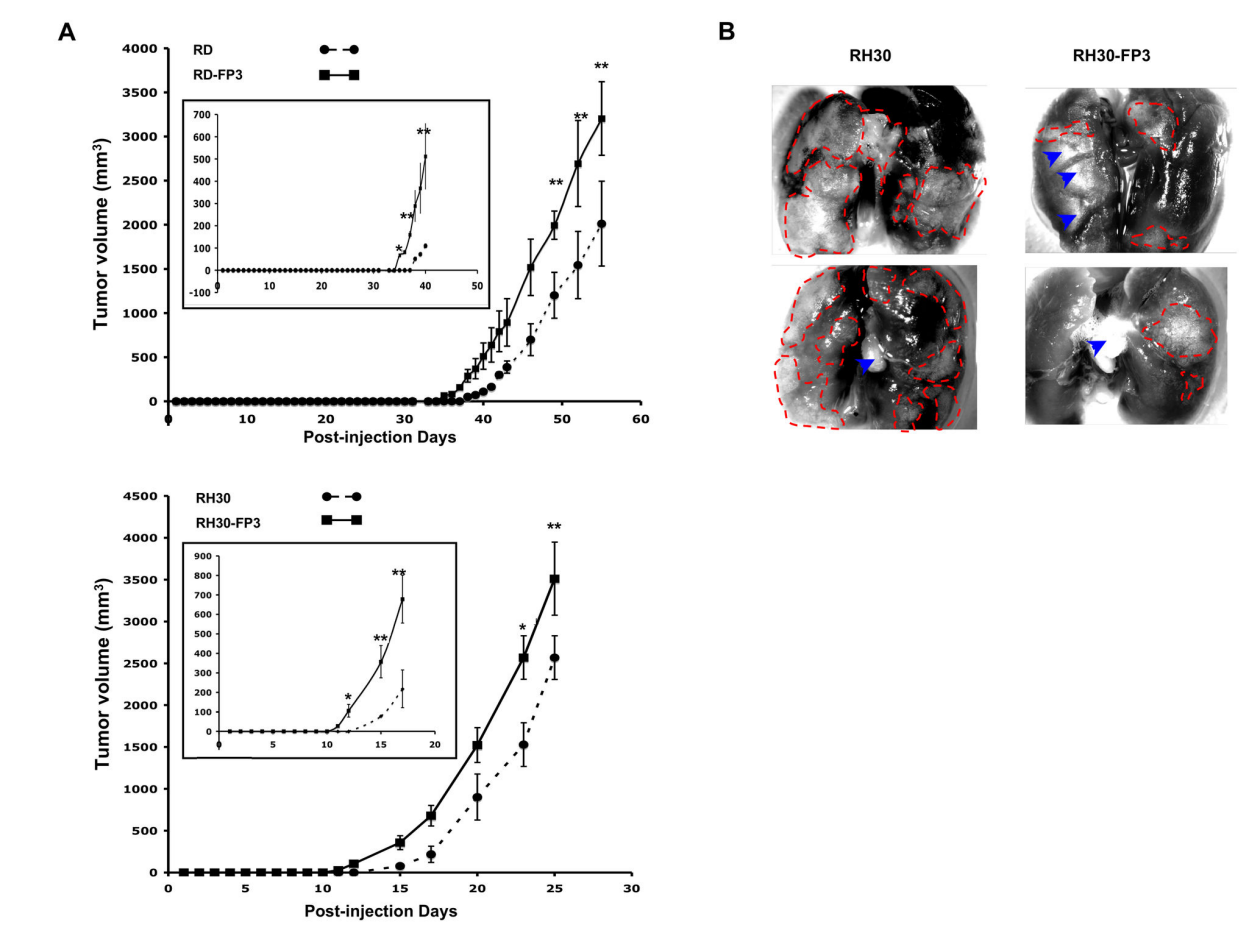
FKHR-PAX3 induced early onset of tumor formation in nude mice xenograft model. (**A**) RD (top panel) and RH30 (bottom panel) cells expressing empty vector or FKHR-PAX3 were injected intramuscularly into the hind legs of nude mice as described in Materials and Methods. Data points represent mean ± s.d. of tumor volume (mm^3^) of all injected mice at the indicted time points. Inset: an expanded view over the early tumor development period. (**B**) Dissecting microscopic images (magnification 25X) of lung organs of two representative mice from the control (left) and FKHR-PAX3 (right) group, showing clear evidence of more extensive tumor mass and infiltration metastasis in the control group (red dashed lines: margins surrounding the tumor mass; blue arrow heads: light-reflective artifacts). Representative data from FKHR-PAX3 isoform c is shown.

In addition, we were not able to detect any measurable transcription activity in transient transfection studies of FKHR-PAX3. Because FKHR-PAX3 retains part of the FKHR DBD, we were most interested in knowing whether FKHR-PAX3 could transactivate an FKHR-responsive promoter such as IGFBP. As shown in Figure 5A, FKHR-PAX3 neither transactivated the IGFBP promoter nor altered the promoter’s response to FKHR (Figure 5A). Even though FKHR-PAX3 helped C2C12 cells maintain high PAX3-FKHR expression, it did not interfere with PAX3-FKHR transcriptional function. We tested three PAX3-FKHR promoters, a PAX3-dependent (e5 [18]) and two PAX3-independent (the paired domain-dependent MyoG [20]; the homeodomain-dependent PDGFαR [18]) promoters. In each case, FKHR-PAX3 did not transactivate these promoters nor did it interfere with their transactivation by PAX3-FKHR (Figure 5 B–D). The lack of transcription activity in FKHR-PAX3 is consistent with its predominant presence in the cytoplasm. These results point to novel mechanistic actions by FKHR-PAX3, which most likely are not shared with PAX3-FKHR and its parent transcription factor PAX3 and FKHR. Of note, we tested all three isoforms of FKHR-PAX3 in our functional (Figures 3-5) and biological studies (Figures 6-9), and found no difference in their behavior.

Whether there can be a role for FKHR-PAX3 in the nucleus or in transcriptional regulation under different circumstances will require additional investigation. Proteins without NLS are known to enter nucleus by alternative mechanisms [42]. FKHR-PAX3 may enter nucleus in response to stimuli that do not normally control FKHR. FKHR-PAX3 clearly can respond differently from FKHR towards the same regulatory signal (Figure 2D). Previous studies with PAX3-FKHR have also shown that this fusion protein can bind to novel DNA sequences that are not recognized by its parent proteins [18,20,21]. Therefore, FKHR-PAX3 could potentially target different promoters than FKHR because it contains only part of the FKHR DBD. There is obviously much work to be done to elucidate the mechanism of FKHR-PAX3 action to explain how it elicits the different biological effects reported below.

### FKHR-PAX3 induced cell proliferation and blocked cell differentiation

We employed a gain-of-function approach to assess the biological properties of FKHR-PAX3. We began by examining the effect of FKHR-PAX3 on proliferation, and found no significant change in the growth rate in any of the cell lines tested (Figure 6A). However, we noticed periodically that if the plated cell density was lower than planned, the FKHR-PAX3 expressing cells appeared to have a faster initial proliferation (e.g., RH4 panel, days 1 and 2). To investigate if this growth burst was real, we deliberately seeded cells at a five-fold lower density, and measured growth rate using the WST-1 proliferation kit to increase sensitivity and accuracy. We found that FKHR-PAX3 consistently accelerated proliferation at low cell density in transformed cells as demonstrated in Figure 6B. This effect of FKHR-PAX3 on low-density growth was not observed in non-transformed NIH-3T3 and C2C12 cells (data not shown).

A major developmental defect in ARMS is the failure to exit cell cycle and complete muscle differentiation, i.e., myotubes formation. To assess whether FKHR-PAX3 blocked myogenesis, we first examined the effect of FKHR-PAX3 on the expression of myogenin (MyoG) and myosin heavy chain (MHC), the respective early and late myogenesis marker, and on the formation of terminally differentiated multinucleated myotubes in a widely used C2C12 myogenic cell model. As shown in Figure 6C, when cultured in differentiation medium, FKHR-PAX3 effectively blocked C2C12 cells from expressing MHC (top panel) and from forming myotubes (bottom panel), but did not prevent these cells from expressing MyoG. PAX3-FKHR has been dubbed a pangene based on its ability to promote early differentiation by directly inducing MyoG expression in undifferentiated cells while simultaneously blocking terminal differentiation [43]. FKHR-PAX3, unlike PAX3-FKHR, did not activate MyoG expression in the absence of differentiation signals, indicating FKHR-PAX3 inhibited myogenesis after cells’ commitment to differentiation. There is evidence that most ARMS cells retain some myogenic potential because they will express MHC and form myotubes upon inhibition of PAX3-FKHR expression, as exemplified in Figure 2D (lanes 1 and 2). The results suggest that the low level of FKHR-PAX3 in RH30 cells is insufficient to block terminal differentiation. Indeed, RH30 cells did not form myotubes when endogenous FKHR-PAX3 was knocked down (data not shown). However, elevation of FKHR-PAX3 protein level through ectopic expression was sufficient to block RH30 cells from terminal differentiation under reduced PAX3-FKHR expression (Figure 6D). This result indicates that the two fusion proteins act independently in blocking myogenic differentiation. This is a particularly important finding because both PAX3-FKHR and FKHR-PAX3 block myogenesis at a similar stage (e.g., MyoG positive) where most ARMS tumor cell differentiation is arrested.

### FKHR-PAX3 promoted colony growth

The effect of FKHR-PAX3 on cell proliferation at low cell density was intriguing as this might reflect its influence on cells’ ability in clonal expansion, a key property of transformed cells. To this end, we measured the effects of FKHR-PAX3 on colony formation using anchorage-dependent clonogenic (Figure 7A) and anchorage-independent soft agar (Figure 7B) growth assays. We found that FKHR-PAX3 promoted colony formation under both assay conditions in RD and RH30 cells. The effect of FKHR-PAX3 on soft-agar colony formation was more pronounced under low (Figure 7B, bottom panel) than high (Figure 7B, top panel) serum conditions. FKHR-PAX3, however, did not promote colony growth in non-transformed cells such as NIH3T3, suggesting that FKHR-PAX3 lacked intrinsic transformation activity. In this regard, FKHR-PAX3 has a more restrictive transforming potential than PAX3-FKHR that promotes robust colony growth in both transformed and non-transformed cells [44–46].

### Effects of FKHR-PAX3 on cell adhesion, mobility, and invasion

We also examined how FKHR-PAX3 affected cell adhesion, migration, and invasion, properties that are critical for tumor progression and metastasis. Cell-matrix assays showed that FKHR-PAX3 enhanced RD and RH30 cell attachment to several extracellular matrix (ECM) proteins such as collagens (I and IV) and laminin (Figure 8A). The FKHR-PAX3 expressing RD and RH30 cells also showed greater resistance to trypsin treatment, an indication of increased adhesion strengths (Figure 8B). Because integrins are major cell surface ECM receptors that signal through focal adhesion kinase (FAK) activation, we examined how FKHR-PAX3 might affect this process by measuring the levels of FAK protein and its phosphorylation status. While FKHR-PAX3 did not alter the total amount of FAK protein, it did increase p-Y397 autophosphorylation, the immediate response of FAK activation cascade to integrin engagement with ECM (Figure 8C). Interestingly, FKHR-PAX3 did not alter the levels of p-Y576, -577, and -925, which are known to be critical for promoting cell movement [47]. This result suggests that FKHR-PAX3 may negatively impact cell motility by stimulating cell adhesion. Indeed, we showed that FKHR-PAX3 expressing RD and RH30 cells were much less motile as demonstrated in the scratch wound (Figure 8D). While the FKHR-PAX3-induced decreases in cell movement through enhanced cell-cell and cell-ECM contacts might be critical during primary tumor formation, these same effects could be counterproductive at later stages such as metastasis. In support of this idea, we found that FKHR-PAX3 reduced the invasiveness of RD and RH30 cells as measured by migration through a Matrigel mix (Figure 8E). The smaller inhibitory effect of FKHR-PAX3 on RD line is likely related to its less aggressive nature of RD cells as evidenced by the longer time needed to assay the scratch wound and Matrigel assays for RD (48 hours) than for RH30 (24 hours).

### FKHR-PAX3 accelerated ARMS xenograft tumor formation in nude mice

Collectively, data from Figures 3 and 6-8 point to a likely role of FKHR-PAX3 that favors tumor induction rather than tumor progression. To further validate this idea, we compared the *in vivo* tumor formation of RD and RH30 cells with or without FKHR-PAX3 following intramuscular injection in nude mice. As shown in Figure 9A, we consistently observed tumor onset about 2-3 days earlier in mice injected with FKHR-PAX3 expressing cells compared to control vector expressing cells (see inset for enlargement of tumor onset). However, FKHR-PAX3 did not affect the overall rate of tumor growth from both cell lines, consistent with the idea that FKHR-PAX3 actively influences the early processes in a developing tumor. All mice within the RD or RH30 groups were sacrificed at the same time and examined for distal metastasis in major organs including brain, lungs, kidneys, and liver. We found metastasis in the lungs of both the RH30 mouse groups but not the RD mouse groups. However, gross examination revealed that the tumor spread (number and size) into the lungs was noticeably less extensive in mice injected with FKHR-PAX3 expressing than control RH30 cells (Figure 9B). This *in vivo* data result is consistent with the *in vitro* effect of FKHR-PAX3 on reducing RH30 cell motility (Figure 8D and 8E). This result further supports the participation of FKHR-PAX3 reciprocal fusion gene in the initiation rather than progression phase of the disease. The negative impact of FKHR-PAX3 on the invasive characteristics offer a plausible explanation to why cancer cells might down regulate FKHR-PAX3 expression as the tumor evolves to become more aggressive. Because our main focus of the xenograft tumor model in this study was to assess FKHR-PAX3’s effect on primary tumor formation, a quantitative assessment of the metastatic process could not be measured in the current experiment. Future study using tail vein injection model with traceable GFP-labeled tumor cells with or without FKHR-PAX3 would be more suitable for testing its effect on tumor metastasis systematically.

## Conclusion

In this report, we present a detailed characterization of FKHR-PAX3 gene products including cloning full-length cDNAs and functional analyses in cellular and mouse tumor models. We show that ARMS cells appropriately process primary transcripts from the FKHR-PAX3 fusion gene as predicted from our knowledge of the translocation sites and the genes involved. The processed transcripts are translated *in vitro* and *in vivo* into proteins of the expected size. Consistent with the prior report using tumor samples, FKHR-PAX3 gene products are not present in all t(2;13) positive ARMS cell lines. When it is expressed, its transcript and protein levels are more comparable to PAX3 and FKHR than to PAX3-FKHR. While it is likely that FKHR-PAX3 is not essential in established cancer cells based on its expression pattern, evidence provided from this investigation points to a role in ARMS tumor initiation. Our conclusion is based on three observations on the ability of FKHR-PAX3 to: (1) enable a sustainable high PAX3-FKHR expression in non-transformed cells, (2) block myogenic differentiation, and (3) promote proliferation under suboptimal growth conditions and induce early tumor formation in mice. First, high levels of PAX3-FKHR are absolutely required for oncogenic transformation and tumorogenesis, yet how precisely the ARMS cells achieve such high levels of PAX3-FKHR remains unknown. Our study offers FKHR-PAX3 as a viable co-oncogenic factor for this purpose. The potential of FKHR-PAX3 to escape negative regulation by PAX3-FKHR might be an important feature at the start of cell transformation process to enable a critical mass of these “transformable starter” cells to accumulate at the site of an emerging tumor. Because FKHR-PAX3 is created at the same time as PAX3-FKHR, the timing is consistent with FKHR-PAX3’s contribution to the early stages of tumorogenesis.

Second, FKHR-PAX3 shows robust inhibitory effects on myogenesis as demonstrated in the C2C12 and PAX3-FKHR knockdown ARMS cell models. Differentiation is a key target in tumor initiation because it acts as a fail-safe device against uncontrolled cell growth. During early phases of tumorogenesis, there is a constant selective pressure at the site of an emerging tumor, which favors cells with survival and proliferative advantages [48,49]. This adaptation process selects for genotypes that promote rapid tumor expansion. The fact that both FKHR-PAX3 and PAX3-FKHR fusion proteins can block myogenic differentiation and promote cell growth is highly significant because they foster a rich environment for the acquisition of additional mutations that contribute to tumorogenesis. During later stages of ARMS development, the cells may become less dependent on the need for FKHR-PAX3 because they have accumulated sufficient levels of genetic mutations to complement PAX3-FKHR in growth and survival.

Third, FKHR-PAX3 can alter a number of growth related properties in cells, particularly under stress conditions such as low cell density (e.g., cell proliferation, colony formation) and low nutrient (e.g., anchorage-independent growth). Additionally, FKHR-PAX3 preferentially supports cell adhesion over migration and invasion. These properties are most critical to early stages of tumorogenesis when a small number of cells escape differentiation and begin the colonization process. It is critical that these cells remain tightly adhered to promote continued proliferation and eventual clonal expansion while cells successively accumulate mutations to further promote tumor growth. The tight cell adhesion may place the tumor cells at a selective disadvantage for metastasis at later tumorogenesis stages, causing cells to lose FKHR-PAX3 either through active gene repression or through extensive genomic rearrangement involving the reciprocal fusion sequence. This notion is supported by our *in vivo* data that shows FKHR-PAX3 expressing RH30 cells start to form tumor much earlier than RH30 control cells, but do not generate lung metastasis as extensively as the control cells.

In closing, the present study is the first to characterize the products and roles of the reciprocal FKHR-PAX3 fusion gene in solid tumor pathogenesis. The *in vitro* and *in vivo* analyses support the notion that FKHR-PAX3 could act as a collaborating partner with the oncogenic PAX3-FKHR fusion protein in tumorogenesis. Because many of the FKHR-PAX3 functions observed in this study work similarly in ERMS and ARMS cells, FKHR-PAX3 could also collaborate with genetic mutations other than PAX3-FKHR. Although FKHR-PAX3 lacks intrinsic transforming ability, it possesses pro-oncogenic functions suggestive of its involvement in the early rather than late stages of ARMS development. This yin-yang role of FKHR-PAX3 might account for its variable expression in the established ARMS samples. The detailed molecular mechanism of FKHR-PAX3 action remains unclear and needs further exploration. The mechanism of action of FKHR-PAX3 is unlikely to be as straightforward as the proven transcription factors PAX3, FKHR and PAX3-FKHR. Future studies are also required to evaluate whether co-expression of FKHR-PAX3 and PAX3-FKHR is sufficient to transform normal cells and induce ARMS tumors in animal models.
